# Determinants of Gross Motor Function in Children With Ambulatory Spastic Cerebral Palsy: A Cross‐Sectional Study in Turkey

**DOI:** 10.1111/jpc.70034

**Published:** 2025-03-18

**Authors:** Atahan Turhan, Merve Kurt‐Aydin, Tülay Tarsuslu

**Affiliations:** ^1^ Department of Physical Therapy and Rehabilitation Kırşehir Ahi Evran University Kırşehir Turkey; ^2^ Department of Physical Therapy and Rehabilitation, Faculty of Health Sciences İzmir Kâtip Çelebi University Türkiye; ^3^ Dokuz Eylül University Department of Physical Therapy and Rehabilitation, Faculty of Physical Therapy and Rehabilitation Türkiye

**Keywords:** cerebral palsy, family characteristics, functional independence, gross motor function, parents

## Abstract

**Aim:**

This study aims to explore the determinants of gross motor function in ambulatory children with spastic cerebral palsy (CP).

**Methods:**

Sixty‐eight children diagnosed with spastic CP type were included in the study. Sociodemographic and clinical information of children with CP and their families was recorded. Children's gross motor function level was classified using the Gross Motor Function Classification System; gross motor function was assessed using the Gross Motor Function Measure‐66 (GMFM‐66); and parental quality of life was assessed using the Paediatric Quality of Life Scale Family Effects Module (PedsQL‐FIM).

**Results:**

No significant differences were observed in gross motor function or parental quality of life between hemiparetic and diparetic CP groups. However, children residing in urban areas showed significantly higher gross motor function and parental quality of life compared to those in rural areas (*p* < 0.05). Moderate correlations were found between gross motor function and physical functioning as well as place of residence (*p* < 0.05). Multiple regression indicated that physical functioning and urban residence were significant predictors of gross motor function, accounting for 37.9% of the variance in the GMFM‐66 score.

**Conclusion:**

This study shows that the quality of life of parents of children and residence in the urban area are independent predictors of gross motor function in children with CP. These findings highlight the importance of considering family well‐being and environmental factors when developing interventions to improve gross motor function outcomes in children with CP.

**Trial Registration:** NCT06439446

**Summary**

- What is already known on this topic ?
  ○ Cerebral palsy (CP) is a neurological disorder caused by brain damage, affecting a person's motor skills, posture, and gross motor functions.
  ○ There are many factors that affect the gross motor function of children with CP.
  ○ One of the factors influencing gross motor function in children with CP is family characteristics, and more evidence is needed to determine their effects on gross motor function.
- What this paper adds ?
  ○ Gross motor function and parental quality of life of children with CP living in urban areas were higher than those living in rural areas.
  ○ It shows that parental quality of life and urban residence of children with CP is independent predictors of gross motor function in children with CP.

## 1 Introduction

Cerebral palsy (CP) is a chronic condition caused by non‐progressive lesions in the developing brain during early childhood, resulting in movement and posture difficulties along with additional issues such as sensory, perceptual, cognitive, and communication disorders [1, 2]. These problems limit the functional activities of children with CP, affecting their activity levels and participation [3]. Among the different types of CP, spastic CP is the most common and represents a significant cause of long‐term disability in children [4, 5].

Given that motor impairments play a central role in limiting activity and participation in children with CP, gross motor function emerges as a key focus of physical therapy and rehabilitation interventions [6]. While motor impairments are a key determinant, gross motor function is also influenced by various environmental and personal factors, highlighting the need for a comprehensive approach to rehabilitation [5, 7]. To effectively address limitations in gross motor function, a holistic approach is essential. Identifying factors associated with gross motor function is crucial for developing and personalising rehabilitation programmes.

Although previous studies have examined various factors influencing gross motor function in children with CP, they often focus on a limited number of determinants, primarily motor impairments or demographic variables [5, 8, 9, 10, 11]. However, these studies often involve diverse CP types and varying ambulation statuses and tend to focus on only one or two relevant factors [5, 9, 10, 11]. However, a more holistic perspective incorporating environmental factors and personal factors within the International Classification of Functioning, Disability and Health (ICF) framework is essential to understanding the multifaceted nature of gross motor function [12, 13]. In children with CP, understanding not only demographic characteristics or factors related to body structure and function but also the impact of social factors, such as place of residence, is crucial for effective process management and the development of appropriate intervention strategies [14]. For instance, individuals living far from healthcare centres may have limited access to certain treatment methods that require repeated applications [13]. In this context, the aim of the present study is to examine the determinants of gross motor function in ambulatory children with spastic type CP.

## 2 Methods

### 2.1 Study Design and Participants

This study was designed as a single‐centre, cross‐sectional, and descriptive investigation, scheduled to take place from June 2023 to August 2024. The study was conducted among children with CP and their families who were being treated at Kırşehir Training and Research Hospital Physical Therapy and Rehabilitation Centre. Children diagnosed with CP and their families who presented to the rehabilitation centre during this period and met the inclusion criteria were enrolled in the study. The research data was collected through face‐to‐face interviews by the researcher (A.T.) at the centre. The sample size was calculated using G*Power 3.1.9.2 software, considering an effect size of 0.35 based on the GMFCS levels from Chen et al.'s study on children with CP [15]. Taking into account an anticipated 10% dropout rate, it was determined that a minimum of 53 participants should be included in the study. The inclusion criteria comprised voluntary participants aged 5 to 15 years diagnosed with spastic hemiparetic or diparetic cerebral palsy, classified at The Gross Motor Function Classification System (GMFCS) levels I, II, and III. Individuals were excluded from participation if they had undergone any surgical procedures within the past year, had contractures in the lower extremities, experienced fractures in the lower extremities within the last six months, received Botulinum Toxin A treatment within the previous six months, or had cognitive, visual, or hearing impairments that could impede effective communication with their peers and siblings.

### 2.2 Procedure

Parents of children meeting the inclusion criteria were informed about the study, and consent was obtained by having them sign an “Informed Consent Form,” which included detailed information about the research. Prior to commencing the study, ethical approval was granted by the Kırşehir Ahi Evran University Non‐Invasive Clinical Research Ethics Committee under decision number 2023–11/72. The research was also registered in the Clinical Trial database system with the identifier NCT06439446.

### 2.3 Outcome Measurements

For the children with CP, age, time to diagnosis, clinical type of CP, gender, weight, height, education level, number of abortions/miscarriages of the children's mother, parents with whom the child lives, number of household members, number of siblings, birth order in the family, education level of the parents, monthly income of the family, per capita income of the family, year of receiving physiotherapy, place of residence with the family, GMFCS level and number of comorbidities were recorded in the evaluation form. GMFCS was used to determine the level of gross motor function in children (disability level), and Gross Motor Function Measure‐66 (GMFM‐66) was used to determine gross motor abilities. The Paediatric Quality of Life Inventory Family Impact Module (PedsQL‐FIM) was used to measure parents' quality of life.

#### 2.3.1 Gross Motor Function Classification System (GMFCS)

The GMFCS is a five‐level classification system used to categorise the motor function abilities of children with cerebral palsy (CP) in activities such as walking, stair climbing, standing, and sitting. Level 1 indicates the highest level of independence, while Level 5 represents the lowest level of independence [16].

#### 2.3.2 Gross Motor Function Measure‐66 (GMFM‐66)

The GMFM‐66 is a scale developed to detect changes in motor functions in individuals with CP aged between 5 months and 16 years. This scale consists of 5 sub‐factors and 66 items. Scoring is done based on a Likert scale, where each item is scored between 0 and 3. The total score is calculated out of 100 points, with higher scores indicating better gross motor function [17].

#### 2.3.3 Paediatric Quality of Life Inventory Family Impact Module (PedsQL‐FIM)

The PedsQL‐FIM is a 5‐point Likert‐type scale developed to measure the quality of life of parents of children with disabilities. It consists of a total of 8 sub‐factors and 36 items. Items in the scale are scored between 0 and 100, with higher scores indicating better functionality [18].

### 2.4 Statistical Analysis

Statistical analyses were performed using IBM SPSS Statistics 26.0 (SPSS Inc., Chicago, IL, USA). The normality of the data distribution was assessed through visual methods (histograms, probability plots) and analytical tests (Kolmogorov–Smirnov test), confirming that the data followed a normal distribution. Descriptive statistics were presented as means ± standard deviation (X ± SD) along with minimum and maximum values for continuous numerical variables, while categorical variables were reported as counts and percentages (%). To compare the gross motor function of children with CP and their parents' quality of life, the statistical analysis used Independent Samples *t* test for two‐group comparisons under parametric conditions, and Mann–Whitney *U* test for two‐group comparisons under non‐parametric conditions. For comparisons involving three groups, Kruskal–Wallis variance analysis was employed. In cases where a significant difference was found in the Kruskal–Wallis variance analysis, Bonferroni correction was applied to determine which evaluations caused the difference. The comparisons were based on the child's diagnosis, place of residence, and the parent with whom the child resides. The correlation between the two continuous variables was analysed by Pearson correlation analysis. Dummy variables were created to include categorical independent variables in the regression analysis. Pearson correlation coefficients were categorised as follows: 0.00–0.10 indicating a negligible correlation, 0.10–0.39 reflecting a weak correlation, 0.40–0.69 indicating a moderate correlation, 0.70–0.89 representing a strong correlation, and 0.90–1.00 reflecting a very strong correlation [19]. Stepwise multiple regression analysis was conducted to identify the determinants of gross motor function. A statistical significance level of *p* < 0.05 was established.

## 3 Results

The study included 68 children diagnosed with CP with a mean age of 9.22 ± 3.08 years; 48.52% of the participants were diparetic CP, while 51.47% were diagnosed with hemiparetic CP. Additionally, 42.64% of the participants were classified as GMFCS Level I. The sociodemographic characteristics of the participants are summarised in Table 1.

**TABLE 1:** Sociodemographic characteristics of participants.

*N* = 68	Mean ± SD or frequency	Min–Max
Age (years)	9.22 ± 3.080	5–15
Time to diagnosis (months)	18.40 ± 13.312	6–60
Diagnosis		
Diparetic cerebral palsy (*n*)	48.52 (33)	
Hemiparetic cerebral palsy (*n*)	51.47 (35)	
Gender		
Male % (*n*)	44.11 (30)	
Female % (*n*)	55.88 (38)	
Weight (kg)	29.40 ± 11.453	13–58
Height (cm)	129.88 ± 19.767	98–170
School attended		
Kindergarten % (*n*)	23.52 (16)	
Primary school % (*n*)	29.41 (20)	
Secondary school % (*n*)	13.23 (9)	
High school % (*n*)	4.41 (3)	
No schooling % (*n*)	29.41 (20)	
Number of abortions	1.53 ± 1.263	0–4
Parents the child lives with		
Two parents % (*n*)	82.35 (56)	
Mother % (*n*)	11.76 (8)	
Father % (*n*)	4.41 (3)	
Grandparents % (*n*)	1.47 (1)	
Number of household members	4.25 ± 0.998	3–7
Number of siblings	1.31 ± 1.026	0–5
Birth order in the family	2.10 ± 1.039	1–5
Maternal educational level		
Secondary school % (*n*)	14.70 (10)	
High school % (*n*)	33.82 (23)	
Associate degree % (*n*)	25.00 (17)	
College % (*n*)	20.58 (14)	
Postgraduate % (*n*)	5.88 (4)	
Paternal educational level		
Secondary school % (*n*)	4.41 (3)	
High school % (*n*)	8.82 (6)	
Associate degree % (*n*)	29.41 (20)	
College % (*n*)	30.88 (21)	
Postgraduate % (*n*)	26.47 (18)	
Family monthly income (TL)	19 764 ± 11964.497	8500–58 000
Per capita income (TL)	4376 ± 2679.611	1800–14 500
Year of receiving physiotherapy	6.43 ± 3.352	1–13
Place of residence		
Urban % (*n*)	52.94 (36)	
Township % (*n*)	33.82 (23)	
Rural % (*n*)	13.23 (9)	
GMFCS		
Level I % (*n*)	42.64 (29)	
Level II % (*n*)	41.17 (28)	
Level III % (*n*)	16.17 (11)	
Number of comorbidities	3.26 ± 2.537	0–6

Abbreviations: GMFCS, Gross Motor Function Classification System; SD, standard deviation; TL, Turkish lira.

No significant difference was found in the gross motor function of children with hemiparetic and diparetic CP or in their parents’ quality of life scores (*p* > 0.05, Table 2). The gross motor function and parental quality of life scores were significantly higher for children living in urban areas compared to those living in rural areas (*p* < 0.05, Tables 2 and 3). Children living with both parents and those living with a single parent or grandparents showed similar gross motor function and parental quality of life (*p* > 0.05, Table 2).

**TABLE 2:** Comparison of functional level and quality of life findings according to diagnosis, place of residence, and living together.

		PedsQL‐FIM	GMFM‐66
		Mean ± SD (Min–Max)	Mean ± SD (Min–Max)
Diagnosis	Diparetic CP (*n* = 33)	49.922 ± 9.501	43.698 ± 5.563
	Hemiparetic CP (*n* = 35)	53.622 ± 7.364	42.538 ± 6.944
	*p* ^a^	0.281	0.527
Place of residence	Urban (*n* = 36)	54.146 ± 7.484	46.174 ± 3.035
	Township (*n* = 23)	51.794 ± 7.328	40.684 ± 6.801
	Rural (*n* = 9)	42.632 ± 10.422	36.985 ± 7.920
	*p* ^b^	0.001**	0.000*
Parents the child lives	Two parents (*n* = 56)	51.711 ± 8.888	42.462 ± 6.658
	Mother (*n* = 8)	51.428 ± 8.987	45.413 ± 3.119
	Father (*n* = 3)	54.345 ± 3.843	47.833 ± 1.345
	Grandparents (*n* = 1)	53.950	46.200
	*p* ^b^	0.954	0.313

Abbreviations: CP, cerebral palsy; GMFM‐66, Gross Motor Function Measure‐66; PedsQL‐FIM, Paediatric Quality of Life Inventory Family Impact Module; SD, standard deviation.

^a^ Independent sample *t* test results.

^b^ Kruskal‐Wallis variance results.

* *p* < 0.001.

** *p* < 0.01.

**TABLE 3:** Multiple comparison of functional level and quality of life findings by place of residence.

Variables	(I) Place of residence	(J) Place of residence	Difference between means (I − J)	SE	*p* ^a^
GMFM‐66	Urban	Township	5.490	1.420	0.001*
		Rural	9.189	1.983	0.000*
	Township	Urban	−5.490	1.420	0.001*
		Rural	3.699	2.092	0.218
	Rural	Township	−3.699	2.092	0.218
		Urban	−9.189	1.983	0.000*
PedsQL‐FIM	Urban	Township	2.352	2.096	0.536
		Rural	11.514	2.928	0.001*
	Township	Urban	−2.352	2.096	0.536
		Rural	9.161	3.088	0.016*
	Rural	Urban	−11.514	2.928	0.001*
		Township	−9.161	3.088	0.016*

Abbreviations: GMFM‐66, Gross Motor Function Measure‐66; PedsQL‐FIM, Paediatric Quality of Life Inventory Family Impact Module; SE, standard error.

^a^ Bonferroni correction analysis results.

* *p* < 0.017.

A moderate correlation was found between the gross motor function of the children included in the study and the physical functioning subscale of the PedsQL‐FIM (*r* = 0.572, *p* < 0.01) as well as between gross motor function and urban residence (*r* = 0.522, *p* < 0.01). There was a weak correlation between gross motor function and family relationships (*r* = 0.276, *p* < 0.05) as well as between gross motor function and the total score (*r* = 0.376, *p* < 0.01). The relationship between family characteristics and the gross motor function of children with CP and parents' quality of life outcomes is shown in Table S1.

The results of the multiple regression analysis showed that physical functioning (a subscale of the PedsQL‐FIM) and urban residence were significant and independent predictors of gross motor function in children with CP, explaining 37.9% of the variance in the GMFM‐66 score. The regression equation was derived as follows: 30.085 + (0.280 × Physical Functioning) + (3.883 × Place of Residence). The results of the regression analysis are presented in Table 4.

**TABLE 4:** Determinants of functional level in children with cerebral palsy.

Dependent variable	Independent variables	B	SE	β	t	VIF	*p*
GMFM‐66	Constant	30.085	2.739	—	10.984	—	0.000
	Physical functioning (PedsQL‐FIM Subdimension)	0.280	0.076	0.413	3.684	1.356	0.000
	Place of residence urban	3.883	1.402	0.310	2.769	1.356	0.007
	R = 0.631; R^2^ = 0.398; Adjusted R^2^ = 0.379 (F = 21.486, *p* = 0.000)						

Abbreviations: B, under standardised regression coefficient; SE, standard error; VIF, variance inflation factors; PedsQL‐FIM, Paediatric Quality of Life Inventory Family Impact Module; GMFM‐66, Gross Motor Function Measure‐66.

## 4 Discussion

The aim of this study was to examine the determinants of gross motor function in ambulatory children diagnosed with spastic‐type CP. The main results showed that the place of residence and the quality of life of the parents explained 37.9% of the variance in gross motor function of children with CP. Living in the city centre and higher levels of parental quality of life were associated with higher gross motor function scores.

In the present study, no significant difference was found in terms of total quality of life score between the parents of children diagnosed with hemiparetic and diparetic CP. However, Romeo et al. reported that mothers of children with hemiparetic CP had the lowest scores in both physical and psychological quality of life, likely due to the behavioural problems of their children [20]. The discrepancy between the two studies may be due to the present study's lack of focus on children's behavioural issues, which could impact family quality of life, as well as the similar distribution of gross motor function scores among hemiparetic and diparetic children, which are closely linked to family quality of life [21, 22].

The gross motor function of children with CP has been shown to be related to the quality of life of their parents. Increased gross motor function in children corresponds with an improvement in parents' quality of life. Guillamón et al. reported that parents of children with CP generally experience lower levels of quality of life and mental health [23]. Several studies comparing children with CP with varying degrees of motor impairments have indicated that parents of children with more severe disabilities report poorer health status than those with less severe disabilities [21, 22]. Horwood et al. stated that the challenges faced by children with CP can lead to motor dysfunction, further exacerbating limitations in daily living activities and social participation, ultimately diminishing the quality of life for both the child and their family [24]. Farajzadeh et al. found that as the level of disability in children with CP increases, the quality of life of caregiving mothers declines [25]. Based on the results of the current study and existing literature, it is thought that the gross motor function of children with CP are closely related to the quality of life of their families, and interventions aimed at improving the gross motor function of children in this population may not only enhance the children's gross motor function but also support the quality of life of their families.

The residence of children and the quality of life of their parents were identified as significant and independent predictors of gross motor function in children with spastic CP. The gross motor function of children with CP and the quality of life of their parents showed significant differences based on their place of residence. It was observed that children living in urban areas had higher gross motor function, and their parents had a better quality of life. Anderson et al. have found that individuals living in rural areas had poorer health outcomes compared to those residing in urban centres [26]. Similarly, Hammal et al. highlighted the impact of living location on the physical dependency, participation, and social interaction of children with CP, noting that factors such as CP type, intellectual disability, and walking ability were influenced by where the child lived [8]. Jahan et al. reported a higher prevalence of CP cases in rural areas, along with inadequate access to rehabilitation services [27]. Schaible et al. also noted that families of children with CP living in rural settings received less healthcare compared to their urban counterparts [28]. Previous studies conducted in Turkey have shown that access to healthcare services varies across regions, with less developed areas facing more significant limitations. As a result, health outcomes also differ accordingly, with individuals in rural areas being at a disadvantage in terms of healthcare accessibility. Although there have been improvements in healthcare services in Turkey, urban areas still offer more favourable access compared to rural regions [29, 30, 31, 32]. A study conducted in Turkey has indicated that living in a rural area is associated with lower quality of life in individuals with disabilities [31]. In line with the existing literature, the present study found that the place of residence significantly affected both the quality of life of families and the gross motor function of children with CP. This may be attributed to the limited access to healthcare services and social support in rural areas, which can create substantial challenges for children with CP and their families. Davis et al. emphasised that caring for a child with CP is a challenging and prolonged process, and insufficient healthcare services can negatively affect parental quality of life [33]. Dagenais et al. stressed the importance of support services for children with CP, highlighting that these services increase parental satisfaction [34]. Given the findings of these studies, it is evident that limited access to healthcare in rural areas is a critical factor affecting both gross motor function in children and the quality of life for their families. Therefore, developing supplementary support methods and strategies through online platforms could be crucial for children with CP and their families living in rural regions. Online services could help bridge the gap in healthcare and social support, providing necessary resources to improve both functional outcomes and overall well‐being.

Our study has limitations. The cross‐sectional design of this study is a limitation that prevents the establishment of a cause‐effect relationship. Additionally, while this study identifies the place of residence as a determinant of gross motor function, the impact of rural residence on access to healthcare services may vary between countries. Differences in healthcare infrastructure, resource distribution, and policies across countries could influence the extent to which living in a rural area affects motor function outcomes in children with CP. Therefore, our findings may not be directly generalisable to children with CP in other countries. Additionally, a limitation of this study is that environmental factors were only assessed based on the place of residence (urban vs. rural). However, environmental factors influencing motor function in children with CP are multifaceted and include aspects such as access to rehabilitation services, availability of assistive devices, transportation difficulties, social support, educational opportunities, and home environment adaptations. Future studies should incorporate a more detailed evaluation of these environmental determinants to better understand their impact on gross motor function in children with CP.

In conclusion, this study shows that the quality of life of parents of children with CP and residence in the urban area is an independent predictor of child gross motor function as assessed by the GMFM‐66. Clinically, these findings highlight the importance of considering family well‐being and environmental factors when developing interventions to improve gross motor function outcomes in children with CP, suggesting that comprehensive approaches that address both child and family needs may be more effective.

## Author Contributions

The author takes full responsibility for this article.

## Conflicts of Interest

The authors declare no conflicts of interest.

## Supporting information


**Table S1.** Correlation analysis between dependent and independent variables.
