# Supplementary material for: Determinants of Gross Motor Function in Children With Ambulatory Spastic Cerebral Palsy: A Cross‐Sectional Study in Turkey
Source: J Paediatr Child Health. 2025 Mar 18;61(5):795–801. doi: 10.1111/jpc.70034 (PMC12053074; doi:10.1111/jpc.70034)
Supplement: Supplementary file 1 — Table S1. Correlation analysis between dependent and independent variables. [file JPC-61-795-s001.docx]

|  |  | 1 | 2 | 3 | 4 | 5 | 6 | 7 | 8 | 9 | 10 | 11 | 12 | 13 | 14 | 15 | 16 | 17 | 18 | 19 | 20 |
| --- | --- | --- | --- | --- | --- | --- | --- | --- | --- | --- | --- | --- | --- | --- | --- | --- | --- | --- | --- | --- | --- |
| 1. GMFM-66 | r | 1 | -0.041 | 0.076 | 0.138 | 0.070 | 0.068 | -0.137 | **0.572^**^** | 0.218 | 0.184 | 0.207 | 0.230 | 0.183 | **0.248^*^** | **0.276^*^** | **0.376^**^** | **0.522^**^** | 0.103 | 0.033 | -0.012 |
|  | p |  | 0.738 | 0.537 | 0.260 | 0.568 | 0.581 | 0.266 | 0.000 | 0.074 | 0.132 | 0.091 | 0.059 | 0.135 | 0.042 | 0.023 | 0.002 | 0.000 | 0.405 | 0.787 | 0.924 |
| 2. Number of siblings | r | -0.041 | 1 | 0.084 | 0.041 | -0.088 | -0.082 | 0.200 | -0.210 | **-0.290^*^** | -0.210 | **-0.292^*^** | 0.000 | -0.060 | -0.066 | 0.020 | -0.108 | -0.032 | 0.028 | -0.046 | -0.095 |
|  | p | 0.738 |  | 0.497 | 0.742 | 0.477 | 0.506 | 0.101 | 0.085 | 0.017 | 0.086 | 0.016 | 0.997 | 0.630 | 0.591 | 0.872 | 0.381 | 0.793 | 0.821 | 0.707 | 0.442 |
| 3. Birth order in the family | r | 0.076 | 0.084 | 1 | 0.095 | -0.104 | 0.148 | **-0.239^*^** | 0.039 | 0.135 | 0.163 | 0.134 | 0.195 | 0.138 | 0.052 | -0.052 | 0.149 | 0.114 | -0.014 | 0.011 | 0.079 |
|  | p | 0.537 | 0.497 |  | 0.440 | 0.399 | 0.229 | 0.050 | 0.752 | 0.272 | 0.184 | 0.277 | 0.111 | 0.262 | 0.673 | 0.676 | 0.224 | 0.356 | 0.911 | 0.931 | 0.520 |
| 4. Family monthly income | r | 0.138 | 0.041 | 0.095 | 1 | **0.341^**^** | 0.017 | -0.024 | 0.032 | 0.070 | 0.090 | 0.022 | 0.155 | 0.054 | 0.082 | 0.104 | 0.046 | 0.078 | 0.007 | 0.139 | 0.129 |
|  | p | 0.260 | 0.742 | 0.440 |  | 0.004 | 0.888 | 0.846 | 0.793 | 0.570 | 0.466 | 0.860 | 0.208 | 0.663 | 0.507 | 0.397 | 0.709 | 0.526 | 0.955 | 0.259 | 0.293 |
| 5. Per capita income | r | 0.070 | -0.088 | -0.104 | **0.341^**^** | 1 | 0.027 | -0.164 | 0.076 | 0.073 | 0.033 | 0.023 | 0.105 | 0.124 | 0.040 | -0.026 | 0.052 | 0.080 | -0.034 | 0.039 | -0.111 |
|  | p | 0.568 | 0.477 | 0.399 | 0.004 |  | 0.828 | 0.183 | 0.538 | 0.553 | 0.787 | 0.853 | 0.393 | 0.312 | 0.747 | 0.833 | 0.674 | 0.518 | 0.785 | 0.750 | 0.366 |
| 6. Number of abortions | r | 0.068 | -0.082 | 0.148 | 0.017 | 0.027 | 1 | -0.047 | 0.121 | 0.013 | 0.225 | 0.016 | 0.234 | 0.201 | -0.014 | -0.006 | 0.094 | 0.022 | -0.015 | 0.099 | 0.070 |
|  | p | 0.581 | 0.506 | 0.229 | 0.888 | 0.828 |  | 0.701 | 0.326 | 0.913 | 0.065 | 0.899 | 0.055 | 0.101 | 0.910 | 0.962 | 0.443 | 0.858 | 0.901 | 0.424 | 0.571 |
| 7. Number of household members | r | -0.137 | 0.200 | **-0.239^*^** | -0.024 | -0.164 | -0.047 | 1 | -0.106 | -0.165 | -0.212 | -0.235 | -0.151 | **-0.315^**^** | -0.062 | 0.066 | -0.103 | -0.119 | -0.173 | -0.104 | 0.102 |
|  | p | 0.266 | 0.101 | 0.050 | 0.846 | 0.183 | 0.701 |  | 0.391 | 0.178 | 0.083 | 0.054 | 0.218 | 0.009 | 0.616 | 0.592 | 0.405 | 0.334 | 0.159 | 0.397 | 0.408 |
| PedsQL-FIM |  |  |  |  |  |  |  |  |  |  |  |  |  |  |  |  |  |  |  |  |  |
| 8. Physical functioning | r | **0.572^**^** | -0.210 | 0.039 | 0.032 | 0.076 | 0.121 | -0.106 | 1 | **0.446^**^** | **0.388^**^** | **0.481^**^** | **0.484^**^** | **0.417^**^** | **0.484^**^** | **0.502^**^** | **0.692^**^** | **0.512^**^** | 0.151 | 0.026 | 0.014 |
|  | p | 0.000 | 0.085 | 0.752 | 0.793 | 0.538 | 0.326 | 0.391 |  | 0.000 | 0.001 | 0.000 | 0.000 | 0.000 | 0.000 | 0.000 | 0.000 | 0.000 | 0.219 | 0.833 | 0.911 |
| 9. Emotional functioning | r | 0.218 | **-0.290^*^** | 0.135 | 0.070 | 0.073 | 0.013 | -0.165 | **0.446^**^** | 1 | **0.591^**^** | **0.969^**^** | **0.278^*^** | **0.323^**^** | 0.221 | **0.401^**^** | **0.700^**^** | 0.113 | -0.189 | -0.011 | 0.103 |
|  | p | 0.074 | 0.017 | 0.272 | 0.570 | 0.553 | 0.913 | 0.178 | 0.000 |  | 0.000 | 0.000 | 0.022 | 0.007 | 0.070 | 0.001 | 0.000 | 0.359 | 0.123 | 0.930 | 0.401 |
| 10. Social functioning | r | 0.184 | -0.210 | 0.163 | 0.090 | 0.033 | 0.225 | -0.212 | **0.388^**^** | **0.591^**^** | 1 | **0.667^**^** | **0.554^**^** | **0.678^**^** | **0.284^*^** | **0.338^**^** | **0.682^**^** | 0.215 | **-0.239^*^** | 0.021 | 0.147 |
|  | p | 0.132 | 0.086 | 0.184 | 0.466 | 0.787 | 0.065 | 0.083 | 0.001 | 0.000 |  | 0.000 | 0.000 | 0.000 | 0.019 | 0.005 | 0.000 | 0.079 | 0.050 | 0.866 | 0.231 |
| 11. Cognitive functioning | r | 0.207 | **-0.292^*^** | 0.134 | 0.022 | 0.023 | 0.016 | -0.235 | **0.481^**^** | **0.969^**^** | **0.667^**^** | 1 | **0.324^**^** | **0.394^**^** | **0.266^*^** | **0.414^**^** | **0.720^**^** | 0.114 | -0.175 | 0.001 | 0.113 |
|  | p | 0.091 | 0.016 | 0.277 | 0.860 | 0.853 | 0.899 | 0.054 | 0.000 | 0.000 | 0.000 |  | 0.007 | 0.001 | 0.028 | 0.000 | 0.000 | 0.354 | 0.153 | 0.995 | 0.358 |
| 12. Communication | r | 0.230 | 0.000 | 0.195 | 0.155 | 0.105 | 0.234 | -0.151 | **0.484^**^** | **0.278^*^** | **0.554^**^** | **0.324^**^** | 1 | **0.782^**^** | 0.107 | **0.260^*^** | **0.553^**^** | **0.356^**^** | -0.159 | 0.109 | 0.076 |
|  | p | 0.059 | 0.997 | 0.111 | 0.208 | 0.393 | 0.055 | 0.218 | 0.000 | 0.022 | 0.000 | 0.007 |  | 0.000 | 0.386 | 0.032 | 0.000 | 0.003 | 0.196 | 0.377 | 0.540 |
| 13. Worry | r | 0.183 | -0.060 | 0.138 | 0.054 | 0.124 | 0.201 | **-0.315^**^** | **0.417^**^** | **0.323^**^** | **0.678^**^** | **0.394^**^** | **0.782^**^** | 1 | 0.117 | **0.294^*^** | **0.534^**^** | **0.340^**^** | -0.040 | 0.101 | 0.031 |
|  | p | 0.135 | 0.630 | 0.262 | 0.663 | 0.312 | 0.101 | 0.009 | 0.000 | 0.007 | 0.000 | 0.001 | 0.000 |  | 0.344 | 0.015 | 0.000 | 0.005 | 0.745 | 0.414 | 0.802 |
| 14. Daily activities | r | **0.248^*^** | -0.066 | 0.052 | 0.082 | 0.040 | -0.014 | -0.062 | **0.484^**^** | 0.221 | **0.284^*^** | **0.266^*^** | 0.107 | 0.117 | 1 | **0.439^**^** | **0.581^**^** | 0.076 | 0.188 | 0.043 | 0.007 |
|  | p | 0.042 | 0.591 | 0.673 | 0.507 | 0.747 | 0.910 | 0.616 | 0.000 | 0.070 | 0.019 | 0.028 | 0.386 | 0.344 |  | 0.000 | 0.000 | 0.538 | 0.125 | 0.730 | 0.953 |
| 15. Family relationships | r | **0.276^*^** | 0.020 | -0.052 | 0.104 | -0.026 | -0.006 | 0.066 | **0.502^**^** | **0.401^**^** | **0.338^**^** | **0.414^**^** | **0.260^*^** | **0.294^*^** | **0.439^**^** | 1 | **0.626^**^** | **0.371^**^** | 0.128 | -0.026 | 0.024 |
|  | p | 0.023 | 0.872 | 0.676 | 0.397 | 0.833 | 0.962 | 0.592 | 0.000 | 0.001 | 0.005 | 0.000 | 0.032 | 0.015 | 0.000 |  | 0.000 | 0.002 | 0.298 | 0.836 | 0.843 |
| 16. Total impact score | r | **0.376^**^** | -0.108 | 0.149 | 0.046 | 0.052 | 0.094 | -0.103 | **0.692^**^** | **0.700^**^** | **0.682^**^** | **0.720^**^** | **0.553^**^** | **0.534^**^** | **0.581^**^** | **0.626^**^** | 1 | **0.288^*^** | -0.061 | 0.049 | 0.076 |
|  | p | 0.002 | 0.381 | 0.224 | 0.709 | 0.674 | 0.443 | 0.405 | 0.000 | 0.000 | 0.000 | 0.000 | 0.000 | 0.000 | 0.000 | 0.000 |  | 0.017 | 0.622 | 0.690 | 0.537 |
| 17. Place of residence urban | r | **0.522^**^** | -0.032 | 0.114 | 0.078 | 0.080 | 0.022 | -0.119 | **0.512^**^** | 0.113 | 0.215 | 0.114 | **0.356^**^** | **0.340^**^** | 0.076 | **0.371^**^** | **0.288^*^** | 1 | 0.200 | 0.056 | 0.051 |
|  | p | 0.000 | 0.793 | 0.356 | 0.526 | 0.518 | 0.858 | 0.334 | 0.000 | 0.359 | 0.079 | 0.354 | 0.003 | 0.005 | 0.538 | 0.002 | 0.017 |  | 0.102 | 0.649 | 0.678 |
| 18. Parents the child lives with two parents | r | 0.103 | 0.028 | -0.014 | 0.007 | -0.034 | -0.015 | -0.173 | 0.151 | -0.189 | **-0.239^*^** | -0.175 | -0.159 | -0.040 | 0.188 | 0.128 | -0.061 | 0.200 | 1 | 0.062 | -0.075 |
|  | p | 0.405 | 0.821 | 0.911 | 0.955 | 0.785 | 0.901 | 0.159 | 0.219 | 0.123 | 0.050 | 0.153 | 0.196 | 0.745 | 0.125 | 0.298 | 0.622 | 0.102 |  | 0.618 | 0.544 |
| 19. Paternal educational level bachelor’s degree | r | 0.033 | -0.046 | 0.011 | 0.139 | 0.039 | 0.099 | -0.104 | 0.026 | -0.011 | 0.021 | 0.001 | 0.109 | 0.101 | 0.043 | -0.026 | 0.049 | 0.056 | 0.062 | 1 | 0.128 |
|  | p | 0.787 | 0.707 | 0.931 | 0.259 | 0.750 | 0.424 | 0.397 | 0.833 | 0.930 | 0.866 | 0.995 | 0.377 | 0.414 | 0.730 | 0.836 | 0.690 | 0.649 | 0.618 |  | 0.300 |
| 20. Maternal educational level high school degree | r | -0.012 | -0.095 | 0.079 | 0.129 | -0.111 | 0.070 | 0.102 | 0.014 | 0.103 | 0.147 | 0.113 | 0.076 | 0.031 | 0.007 | 0.024 | 0.076 | 0.051 | -0.075 | 0.128 | 1 |
|  | p | 0.924 | 0.442 | 0.520 | 0.293 | 0.366 | 0.571 | 0.408 | 0.911 | 0.401 | 0.231 | 0.358 | 0.540 | 0.802 | 0.953 | 0.843 | 0.537 | 0.678 | 0.544 | 0.300 |  |

**Suplementary Table 1.** Correlation Analysis Between Dependent and Independent Variables

PedsQL-FIM: Pediatric Quality of Life Inventory Family Impact Module; *: p<0.05; **: p<0.01
